# Naturally Occurring Extracellular Matrix Scaffolds for Dermal Regeneration: Do They Really Need Cells?

**DOI:** 10.1155/2015/839694

**Published:** 2015-10-05

**Authors:** A. M. Eweida, M. K. Marei

**Affiliations:** ^1^Department of Head and Neck Surgery, Faculty of Medicine, University of Alexandria, Alexandria 21111, Egypt; ^2^Department of Plastic & Reconstructive Surgery, University of Heidelberg, 67071 Ludwigshafen, Germany; ^3^Tissue Engineering Laboratories, Faculty of Dentistry, University of Alexandria, Alexandria 21111, Egypt

## Abstract

The pronounced effect of extracellular matrix (ECM) scaffolds in supporting tissue regeneration is related mainly to their maintained 3D structure and their bioactive components. These decellularized matrix scaffolds could be revitalized before grafting via adding stem cells, fibroblasts, or keratinocytes to promote wound healing. We reviewed the online published literature in the last five years for the studies that performed ECM revitalization and discussed the results of these studies and the related literature. Eighteen articles met the search criteria. Twelve studies included adding cells to acellular dermal matrix (ADM), 3 studies were on small intestinal mucosa (SIS), one study was on urinary bladder matrix (UBM), one study was on amniotic membrane, and one study included both SIS and ADM loaded constructs. We believe that, in chronic and difficult-to-heal wounds, revitalizing the ECM scaffolds would be beneficial to overcome the defective host tissue interaction. This belief still has to be verified by high quality randomised clinical trials, which are still lacking in literature.

## 1. Introduction

The extracellular matrix (ECM) is a complex mixture of structural and functional proteins, glycoproteins, and proteoglycans arranged in a unique, tissue specific three-dimensional (3D) ultrastructure. The pronounced effect of ECM scaffolds in supporting tissue regeneration is related mainly to two major characteristics: the maintained 3D structure and the bioactive components. Their natural 3D structure provides structural support and tensile strength, attachment sites for cell surface receptors, and a reservoir for signaling factors that modulate angiogenesis, cell migration, cell proliferation, and orientation in wound healing [1]. The bioactive components include but are not limited to collagen, laminin, fibronectin, glycosaminoglycans, and a various group of growth factors (VEGF: vascular endothelial growth factor, bFGF: basic fibroblast growth factor, EGF: epidermal growth factor, TGF-beta: transforming growth factor-beta, KGF: keratinocyte growth factor, HGF: hepatocyte growth factor, and PDGF: platelet derived growth factor). The presence of such bioactive molecules, together with their native inhibitors, in their preserved natural 3D spatial structure provides a very convenient platform for cells to regenerate [1, 2].

**Figure 1:**
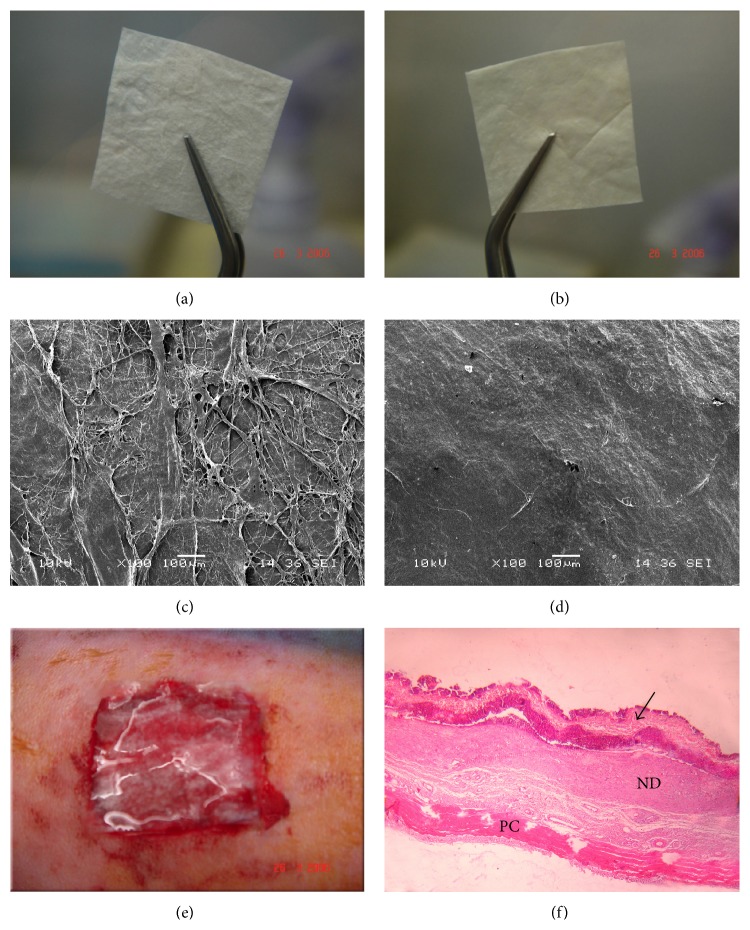
Urinary bladder matrix scaffold. (a) Rough surface. (b) Smooth surface. (c) UBM rough surface (SEM). (d) UBM smooth surface (SEM). (e) Implantation of UBM on full thickness wounds in rabbits (rough surface downwards). (f) H&E section of the wound after 1 week of grafting. Arrow points to the UBM. PC: Panniculus carnosus layer. ND: neodermis. Original magnification ×40.

The decellularized dermis of the skin, submucosa of the small intestine and urinary bladder (Figure 1), and the amniotic membrane are of the commonest sources for ECM scaffolds used for tissue regeneration. Various market products were developed from naturally occurring ECM scaffolds and were approved as wound dressing for skin wounds and burns. Alloderm is one of the first approved acellular matrix materials and was extensively investigated in literature. It is processed directly from fresh cadaver skin that is treated with high salt to remove the cellular components. It is then freeze dried, leaving an immunologically inert acellular dermal matrix with intact basement membrane complex. Approved by the FDA, it has been used to treat burns since 1992. Oasis is a product derived from porcine small intestinal submucosa (SIS). It has been studied at Purdue University in West Lafayette, USA, and is now commercially available as wound dressing [3]. Graft Jacket is a cryogenically stored acellular dermal matrix (ADM) originating from cadaveric skin that is already approved for wound care purposes [4]. Epiflex is a human acellular dermal matrix transplant manufactured from screened consenting donors [5]. Endoform is an approved extracellular matrix created from the submucosa of the sheep fore-stomach, a tissue whose structure is similar to the dermis [6]. MatriStem MicroMatrix (ACell, Columbia, MD, USA) is a recently approved UBM scaffold for wound regeneration [7]. Although proved beneficial for acute and simple wounds the literature lacks high quality clinical evidences that these scaffolds can provide the desirable effects when applied to chronic, difficult-to-heal wounds.

The pathophysiology of chronic wounds and ulcers is usually too complex to be reversed by adding a single factor or cellular component. Chronic ischemic or diabetic wounds as an example are thought to result from the combined comorbidities of neuropathy, vascular deficits, impaired immunity, infection, and repeated tissue trauma, all overlapping to produce a vicious cycle that is very difficult to break [8]. Standard surgical care of such chronic complicated wounds usually fails to match patient's satisfaction and restore the quality of life, and sometimes very complex surgical procedures are required to treat such wounds [9].

Inhibition of extracellular matrix deposition and increased activity of matrix metalloproteinases (MMPs) with concomitant decreased activity of MMP inhibitors were suggested as mechanisms for delayed wound healing in chronic wounds. Regarding the cellular factors; fibroblasts are usually senescent, keratinocytes show impaired migration, and leukocytes exhibit impaired intracellular killing functions. Recently, an impaired function of the gap junctions has immerged as an additional pathological mechanism leading to impaired wound healing. Associated neuropathy leads to a decreased level of neuropeptides that normally contribute to healing. Neuropathy reduces capillary blood flow and vice versa [10–12]. These complex factors and mechanisms suggest that providing the wound with a new viable “tissue” and “milieu” is mandatory to achieve a significant response.

The ECMs are characterized by early degradation so that a major part of their role depends on the active interaction with the recipient cells and tissue. In difficult-to-heal wounds this interaction is usually defective due to a lack of reaction by recipient cells.

In an attempt to overcome this, a process of introducing cells into the biostatic graft, known as “revitalization,” could help these scaffolds perform their function, at least for the early stage after implantation. The grafted cells are usually the recipient's autologous cells (differentiated or stem cells) that are seeded either directly onto the scaffold or after retrieval and propagation in culture [13]. Revitalization of ECM scaffolds with keratinocytes, fibroblasts, or stem cells were shown to improve vascularization, scaffold integration, and cellular proliferation [14–16]. We reviewed the online published literature in the last five years for the studies that performed ECM revitalization and discussed the result of these studies and the related literature.

## 2. Materials and Methods

A PubMed search was performed for the articles published in English language within the previous 5 years. All the articles related to adding keratinocytes, fibroblasts, or stem cells to naturally occurring ECM scaffolds were included. The following string was used for the online search:

 (urinary bladder matrix OR UBM OR small intestinal mucosa OR SIS OR decellularized skin OR alloderm OR acellular dermal matrix OR oasis OR graftjacket OR endoform OR matristem OR Epiflex) AND (keratinocytes OR fibroblasts OR stem cells) AND (skin regeneration OR skin repair OR skin reconstruction OR wound OR burn) AND (English[lang]) AND (“last 5 years”[PDat] AND (Humans[Mesh] OR Animals[Mesh:noexp]))

## 3. Results

The search string yielded 121 articles. The articles were filtered according to title, abstract, and full text resulting in 18 articles that met the search criteria. Twelve studies included adding cells to ADM, 3 studies were on SIS, one study was on UBM, one study was on amniotic membrane, and one study included both SIS and ADM loaded constructs. All in vivo studies were experimental and no single clinical study was found. The type of the study and the most relevant results and remarks are summarized in Table 1.

## 4. Discussion

Although there are no guidelines that clearly recommend the use of ECM scaffolds for wound healing, their benefit in acute wounds and burns has been demonstrated in several clinical studies. The complex mixture of structural and functional proteins, glycoproteins, and proteoglycans retained in its original 3D structure provides the key benefit of using these scaffolds for wound healing. This structure provides a temporary support into which cells can migrate and proliferate in a well-organized and controlled fashion leading to improved wound healing. The suggested mechanisms of wound improvement when applying the ECM scaffolds alone are related to providing a structural support, stimulating angiogenesis, chemotaxis for endothelial cells, and release of growth factors [17, 18].

In case of chronic and difficult-to-heal wounds the challenge is much bigger. The suggested role of ECM scaffolds in improving such wounds is not fully understood. It has been suggested that they would act as a biological cover that modulates the wound environment by reducing the inflammatory activity to promote wound healing [19]. There is currently limited published data that reaches a sufficient level of evidence about the role of ECM scaffolds alone in chronic and difficult-to-heal wounds [3, 20–27].

The positive role of combining ECM scaffolds with stem cells, fibroblasts, or keratinocytes was clearly demonstrated in in vitro and experimental in vivo studies. It is believed that native stem cells play an important role in wound regeneration or healing. GFP-labelled MSCs were found in the skin of non-GFP mice after peripheral injection. This indicates that wounding stimulates MSCs to migrate via chemotaxis to the injury site and differentiate to functional skin cells [28]. Some studies have indicated that wound healing is enhanced through ADSCs that promote human dermal fibroblast proliferation by direct cell-to-cell contact and via a paracrine effect [29].

However, the relation between the efficacy of wound healing and the number of transplanted MSCs does not seem to be a linear one. Yeum et al. [30] have shown that repeated injection of additional MSCs did not increase the number of MSCs participating in wound healing beyond a certain constant maximum amount. The number of MSCs in the wound site remains constant in the range 2-3 × 10^5^ from day 1 to day 10. MSCs were not detected after day 10, probably because the role of transplanted MSCs ended thereafter. Lam et al. [31] also could not detect the signals after 12 days postwounding. It was suggested that the stem cells would have been engulfed by macrophages or migrated to other body sites speculating that after the completion of the MSCs' roles, the wound site no longer needs the MSCs as it has recovered completely by 14 days.

Although the effect of stem cells is well documented in promoting wound healing, these cells usually do not survive well when directly transplanted to the wound site. Many studies have shown that a great number of cells die during transplantation and this effect would be diminished if cells were allowed to proliferate in an optimal milieu [32, 33]. Attempts for aiding stem cell survival often involve codelivery with slow release and survival-promoting gels such as Matrigel or collagen gel. In several in vivo and in vitro studies Matrigel was found to be superior probably due to its basement membrane component [34–36]. Similar studies on SIS have demonstrated that the ECM patch allowed the stem cells to remain localized to the wound area rather than migrate to other regions as evidenced by in vivo cell tracking [31].

Orbay et al. [37] concluded that ADSCs could attach to ADM and decrease its in vivo resorption suggesting that this construct may be a useful tool for soft tissue augmentation with stable long-term results. This effect was thought to be due to stimulatory effects of ADSCs on fibroblasts leading to an indirect increase in the synthesis of collagen and extracellular matrix components.

In an attempt to enhance wound epithelialization, keratinocytes were added to ECM scaffolds in various studies. Based on the in vitro behaviour of the keratinocytes, Zajicek et al. [38] suggested that the ADM promotes wound healing through supporting the growth of patient's own keratinocytes from the adnexa remnants in the wound by providing optimal conditions for their attachment, proliferation, and migration. Peramo et al. [39] proved that Alloderm could also permit the differentiation and stratification of nonkeratinized, buccal mucosa in vitro.

Regarding their effect on the dermal regeneration, Seland et al. [40] have shown that implantation of a single cell layer of keratinocytes to the ADM added nothing to the dermal thickness in the wound healing process. Interestingly keratinocytes loaded on microcarriers showed a significantly thicker epithelium and neodermis at both 16 and 21 days after grafting compared to the wounds treated with a single layer. This led to the hypothesis that these carriers could act as a facilitator for the dermal regeneration beside their role in transportation and transplantation of autologous keratinocytes.

For the recipient keratinocytes to proliferate and uniformly stratify above/within the ECM, it was traditionally known that an optimal environment would require the presence of fibroblasts [41]. This is probably due to the paracrine interaction between the two cell types [42, 43]. Deshpande et al. have concluded in their in vitro study, however, that the formation of a well-organized epithelium on the acellular dermal matrix depends mainly on the presence of intact basement membrane but is largely independent of the presence of cultured fibroblasts. They have noticed that incorporating fibroblasts in the absence of a basement membrane had no significant effect on the keratinocyte behavior [44]. Other groups have demonstrated an enhanced keratinocyte migration on a sterilized dermis after removal of basement membrane antigens but in the presence of fibroblasts under conditions of normal extracellular calcium concentration [45]. These conditions probably represent the in vivo situation during normal wound healing, when the basement membrane has been traumatically disrupted and fibroblast numbers are upregulated in order to heal the wound [46]. We guess that the solution for these contradictory results is the establishment of a well-standardized in vivo study for the assessment of the definite role of fibroblasts and basement membrane factors.

In chronic and difficult-to-heal wounds, vascularisation of the wound bed is a major concern. If STSG is to be implanted over the ADM, then adequate scaffold neovascularisation would be an essential prerequisite. Neovascularisation of the matrix occurs during the early stages of complete adherence of ADM to the recipient wound bed [47]. Increasing and accelerating this neovascularisation and estimating its timing are thus important for an optimal treatment plan [48]. An enhanced angiogenesis through the application of ECM scaffolds was also suggested as an important factor in decreasing wound fibrosis [31]. Sahin et al. [48] have demonstrated that adding MSCs to the ADM has a significant positive effect on the vascularisation probably due to enhanced secretion of VEGF [49]. Han et al. [50] have also demonstrated that enhancement of ADM engraftment and wound angiogenesis could be achieved by seeding of microencapsulated VEGF-expressing fibroblasts below the scaffold. Huang et al. [51] have also demonstrated that DiI-labeled cells were colocalized with staining for VEGF and vWF (Von Willebrand factor) well 14 days after seeding on ADM and implantation in full thickness wounds, suggesting that the grafted cells might improve angiogenesis via the indirect paracrine effect or contribute to newly formed vasculature. Our research group has also demonstrated an enhanced angiogenic activity with autologous keratinocyte grafting with porcine UBM, which could be attributed to a cross talk between the keratinocyte and endothelial cells and release of angiogenic factors from UBM degradation, or even from the dying keratinocytes after grafting [52].

In difficult-to-heal wounds as in chronic or irradiated wounds, it is always wise to bring new healthy “tissue” to the wound bed. Applying the same concept makes adding cells to the scaffold crucial for wound regeneration in such difficult situations where the wound regeneration capacity is subnormal. Roessner et al. [15] have demonstrated that adding fibroblasts to ADM in irradiated wounds would improve wound healing evidenced by enhanced wound tensile strength. This effect was abolished when the transplanted cells where irradiated in an adjuvant-radiotherapy setting.

In a clinical setting, these difficult-to-heal wounds were almost exclusively treated with cell-loaded non-ECM scaffolds such as Apligraf, Dermagraft, and GammaGraft [53]. From all the available ECM scaffolds, only the SIS (Oasis) and to a lesser extent Graft Jacket have been reported clinically in a considerable number of patients to improve chronic wounds without adding cells [3, 21, 25]. The role of SIS in promoting wound closure was extensively investigated. Shi et al. [16] have demonstrated that MMPs inhibit keratinocyte migration in vitro and that preincubating the MMP solution with SIS could significantly reduce this inhibitory effect. MMPs are important contributors to wound chronicity and are abundantly expressed in chronic ulcers and not in acute wounds [54]. MMPs inhibit keratinocyte migration and degrade fibronectin, growth factors, and other proteins vital to wound healing and thus reducing elevated levels of MMPs in chronic wounds should promote healing [55].

A high quality randomized controlled clinical study comparing the wound healing potential of cell free versus cell loaded ECM scaffolds is unfortunately still lacking. Lev-Tov et al. [56] have introduced a protocol to compare the standard surgical care either alone or with Dermagraft (bioengineered ECM containing living fibroblasts) or with UBM (Oasis). Although Dermagraft is not a naturally occurring ECM scaffold, the data coming out of such a study would be useful in understanding the relative role of ECM and added cells in a clinical context.

We think that in difficult-to-heal wounds adding cells to the ECM scaffolds would enhance their regenerative capacity. In acute and simple wounds, however, the regenerative capacity of the native tissues are usually preserved so that the high costs and time linked to adding autologous cells within good clinical practice guidelines could be avoided as the relative benefit would be negligible. These conclusions are based on our surgical and experimental experiences and still have to be verified by high quality randomised clinical trials.

## Figures and Tables

**Table 1:** Studies applying cells to ECM scaffolds in the last 5 years.

Research group	Type of the study	ECM and loaded cells	Results	Remarks
Castagnoli et al. 2010 [57]	Noncomparative in vitro study	Human ADM + human keratinocytes	Preparation and characterization of a new cutaneous biosubstitute made up of alloplastic acellular glycerolized dermis & cultured autologous keratinocytes	(i) No in vivo studies (ii) Proof of principle

Han et al. 2010 [50]	Comparative in vivo study	Porcine ADM + autologous STSG +/− microencapsulated VEGF-expressing fibroblasts	Significant increase in survival & microvessels density in grafts containing microencapsulated VEGF-expressing cells	Cells were injected below the ADM and STSG

Eweida et al. 2011 [52]	Comparative in vivo study	Porcine UBM +/− rabbit keratinocytes	Reduction of early wound contraction and improving wound vascularity	(i) Keratinocytes were transplanted on the rough surface of the UBM (ii) No in vivo cell tracking

Liu et al. 2011 [14]	Comparative in vivo study	Mouse ADSC +/− porcine SIS +/− porcine ADM	Cell loaded ECM scaffolds showed better angiogenesis and early wound closure than cell-free ECM and cell loaded non-ECM scaffolds	The study emphasised the synergistic effect of ECM scaffolds and ADSC on angiogenesis

Lugo et al. 2011 [58]	Noncomparative in vivo study	Human ADM + human keratinocytes	The prevascularized neodermis supported the transplanted keratinocytes leading to a superior wound epithelialization	Keratinocytes were added in fibrin gel one week after implantation of the angiogenic factors-infiltrated ADM

Orbay et al. 2011 [37]	Comparative in vivo study	Rat ADM +/− rat ADSC	The construct enhanced the volume maintenance, vascular density, and collagen content in a subcutaneous soft tissue augmentation model in rats	The SC augmentation model did not address wound healing aspects related to epithelialization

Roessner et al. 2011 [15]	Comparative in vivo study	Human ADM (Epiflex) +/− rat fibroblasts +/− irradiation	Fibroblasts added no significant difference regarding soft tissue volume regeneration. However, a significant increase in wound tensile strength was noted if the transplanted cells were not subjected to irradiation	(i) The ADM was implanted within a deeper tissue defect to replace excised muscles (ii) Due to this special defect design, the increase in wound breaking strength may not be directly related to the physical presence of the seeded implants

Seland et al. 2011 [40]	Comparative in vivo study	Human ADM +/− human keratinocytes (loaded on microcarriers or as single layer or as STSG)	Only the keratinocytes implanted as STSG or loaded on microcarriers had a significant positive effect on epidermal and dermal thickness at 16 & 21 days after transplantation	(i) Keratinocytes were added to the fibrin pretreated wounds fourteen days after the initial transplantation of ADM (ii) In vivo tracking of transplanted cells was performed till the end of the experiment

Huang et al. 2012 [51]	Comparative in vivo study	Mouse ADM +/− human ADSCs	Increased thickness of granulation tissue, improved reepithelialization & wound closure rate, and increased vascular density	(i) ADSCs were seeded on ADM and not directly to the wound bed (ii) In vivo cell tracking was performed till day 14 (iii) VEGF-expressing ASCs could be detected after transplantation

Peramo et al. 2012 [39]	Noncomparative in vitro study	Human ADM (Alloderm) + human keratinocytes (from skin and oral mucosa origins)	In vitro development of human mucocutaneous lip junction equivalent	(i) In vitro proof of principle and was not examined in vivo (ii) Maintaining this delicate transition zone would be challenging in a normal surgical setting

Shi et al. 2012 [16]	Noncomparative in vitro study	SIS + human keratinocytes in a high MMP medium	SIS inhibits the MMP activity and thus promotes keratinocyte migration	The study focuses on the role of the bioactive structure of SIS rather than its scaffolding properties

Zajicek et al. 2012 [38]	Noncomparative in vitro study	Porcine ADM (Xe-Derma) + human keratinocytes	The results suggest that the firm natural structure of ADM stimulates proliferation and differentiation of human primary keratinocytes	A concomitant in vivo study involved the application of only the scaffold without adding cells in acute wounds

Deshpande et al. 2013 [44]	Comparative in vitro study	Human ADM + keratinocytes +/− fibroblasts +/− basement membrane	The formation of a well-organized epithelium depends on the presence of intact basement membrane but is independent of the presence of cultured fibroblasts	Exclusively in vitro study

Huang et al. 2013 [59]	Comparative in vivo study	Human keratinocytes +/− cross-linked human acellular amniotic membrane	Combination of keratinocytes with the acellular amniotic membrane significantly reduced wound contraction at 4 weeks than the cells alone	The study did not include a group with the ECM alone

Lam et al. 2013 [31]	Comparative in vivo study	+/− mouse ADSC +/− porcine SIS	(i) In vivo cell tracking revealed a significant increase in stem cell survival and proliferation with SIS (ii) Delivering stem cells on the SIS significantly decreased fibrosis but slightly improved healing, while SIS alone hindered healing as the patch stented the wound open	(i) A splinted excisional wound model was used to simulate human wound healing and minimize healing by contracture (ii) The special splint-wound design and the too early removal of the SIS patch in some groups (2 days) led to unfavorable results in terms of wound healing

Sahin et al. 2013 [48]	Comparative in vivo study	Human ADM +/− rat bMSCs	Increased, adherence, angiogenesis, and vertical vascular penetration of ADM especially if combined with negative pressure dressing therapy	(i) The MSCs were added once & randomly to the wound bed before ADM implantation (ii) The bMSCs were not tracked in vivo (iii) The early adherence of ADM was probably related to early angiogenesis

Yeum et al. 2013 [30]	Comparative in vivo study	SIS +/− mouse bMSCs	Enhanced wound closure and less wound inflammation with bMSCs	(i) bMSCs were repeatedly transplanted every 2 days for 2 weeks (ii) In vivo cell tracking was performed

Bondioli et al. 2014 [60]	Comparative in vitro study	Fibroblasts +/− human ADM	The matrix extract significantly increased the proliferation rate of fibroblasts	Only an in vitro study as part of the characterization of the matrix

ADSC: adipose derived stem cells.

bMSC: bone marrow derived stem cells.

STSG: split thickness skin graft.

## List of Abbreviations

ADM: Acellular dermal matrix
ADSC: Adipose derived stem cells
bFGF: Basic fibroblast growth factor
bMSC: Bone marrow derived mesenchymal stem cells
EGF: Epidermal growth factor
HGF: Hepatocyte growth factor
KGF: Keratinocyte growth factor
MMP: Matrix metalloproteinases
PDGF: Platelet derived growth factor
STSG: Split thickness skin graft
TGF-beta: Transforming growth factor-beta
UBM: Urinary bladder matrix
VEGF: Vascular endothelial growth factor
vWF: Von Willebrand factor.
